# Bacterial and fungal communities in chronic rhinosinusitis with nasal polyps

**DOI:** 10.1371/journal.pone.0304634

**Published:** 2024-05-31

**Authors:** Eray Uzunoğlu, Ayşe Kalkancı, Esra Kılıç, Yusuf Kızıl, Utku Aydil, Kadir Serdar Diker, Süleyman Sabri Uslu

**Affiliations:** 1 Department of Otorhinolaryngology, Izmir Ekol Hospital, İzmir, Turkey; 2 Department of Medical Microbiology, Gazi University Hospital, Ankara, Turkey; 3 Department of Otorhinolaryngology, Gazi University Hospital, Ankara, Turkey; 4 Department of Microbiology, Adnan Menderes University Faculty of Veterinary Medicine, Aydin, Turkey; University of Pittsburgh, UNITED STATES

## Abstract

**Objective:**

Multiple inflammatory mechanisms dynamically interact in the development of chronic rhinosinusitis with nasal polyps (CRSwNP). Disruption of the relationship between host and environmental factors on the mucosal surface leads to the development of inflammation. Microorganisms constitute the most important part of environmental factors.

**Methods:**

28 volunteers (18 CRSwNP patients and 10 healthy individuals) were included in the study. Eight patients were recurrent nasal polyposis cases, and the remaining were primary cases. Swab samples were taken from the middle meatus under endoscopic examination from all participants. After DNA extraction, a library was created with the Swift Amplicon 16S + ITS kit and sequenced with Illumina Miseq. Sequence analysis was performed using QIIME, UNITE v8.2 database for ITS and Silva v138 for 16S rRNA.

**Results:**

The predominant bacteria in all groups were Firmicutes, Proteobacteria, Actinobacteria as phyla *and Staphylococcus*, *Corynebacterium*, *Sphingomonas* as genera. Comparison of bacterial communities of CRSwNP patients and control group highlighted *Corynebacterium*, as the differentiating taxa for control group and Streptococcus, Moraxella, Rothia, Micrococcus, Gemella, and Prevotella for CRSwNP patients. The predominant fungal genus in all groups was *Malassezia*. Staphylococcus; showed a statistically significant negative correlation with *Dolosigranulum*. Corynebacterium had a positive correlation with *Anaerococcus*, and a negative correlation with *Neisseria*, *Prevotella*, *Fusobacterium* and *Peptostreptococcus*.

**Conclusion:**

Nasal microbiome of CRSwNP patients shows greater inter-individual variation than the control group. Corynebacterium is less abundant in patients with CRSwNP compared to the control group. *Malassezia* is the predominant fungus in the nasal cavity and paranasal sinuses and correlates positively with the abundance of *Corynebacterium*.

## Introduction

Chronic rhinosinusitis with nasal polyposis (CRSwNP) is a complex disease with multifactorial development. Although its etiology has not been fully clarified, it is known that more than one inflammatory mechanism interacts dynamically in the development of the disease. These inflammatory mechanisms are thought to be result from the altered relationship between host and environmental factors at the mucosal surface. Microorganisms constitute the most important part of environmental factors [1].

The human microbiome is essential for development of immunity and maintaining health [2, 3]. Microbiome research has gained momentum with the advances in DNA sequencing methods and the ability to identify microorganisms at the genome level [4]. Nasal microbiome and its relation to diseases have also become popular research topics. Opportunistic pathogens are found in the nasal microbiome and can spread to the other parts of the respiratory tract under favorable conditions and contribute to the development of diseases such as allergic rhinitis, chronic rhinosinusitis, asthma, otitis media [5, 6]. Commensal bacteria in the nasal cavity prevent opportunistic pathogen colonization by competing for limited space and nutrients or by producing toxins [7]. Dysbiosis is defined as compositional or functional difference in the microbiome, compared to the microbiome of healthy population and may occur as decrease in beneficial microorganisms, increase in potential pathological microorganisms or decrease in total microbial diversity [8]. Although the relationship between CRS and a specific bacterium has not been established, dysbiosis plays a role in the pathogenesis of the disease [9].

The role of microbial factors in the pathogenesis of chronic rhinosinusitis with nasal polyps has not been clarified yet and is still an important research topic. In this study, the difference in the bacterial and fungal composition of the nasal microbiome between the healthy control group and CRSwNP patients was investigated. In addition, microbiome of patients who had previously undergone functional endoscopic sinus surgery (FESS) for the treatment of CRSwNP and whose disease had recurred and those who were not surgically treated were compared to each other. Bacterial and fungal populations that could be associated with disease severity were also investigated.

In this study we used a novel method as 16S rRNA and ITS next-generation sequencing on the same sample with a single kit in CRSwNP patients. This made a significant contribution to our study in terms of evaluating the bacterial-fungal interaction. In addition, sequencing of all variable regions between the V1-V9 regions of 16s rRNA increased the power of the study.

## Materials and methods

### Study population

Adult patients diagnosed with CRSwNP in Gazi University Department of Otorhinolaryngology between July 2020 and December 2020 were included in the study following Gazi University Clinical Research Ethics Committee approval (Approval date: 22.05.2020 Approval number: 350). A written informed consent was obtained from all participants.

### Participants’ characteristics

Exclusion and inclusion criteria were as the follows: patients with cystic fibrosis, primary ciliary dyskinesia, patients who used antibiotics and intranasal corticosteroids in the last 4 weeks, and patients who had an additional disease or drug use that could cause immunosuppression were excluded from the study.

It was planned to include two patient groups and a control group in the study. The first patient group consists of CRSwNP patients who have not been treated with FESS (primary disease group). The second group of patients consisted of those who had previously undergone FESS due to CRSwNP and who had a recurrence of the disease (recurrent disease group). SNOT-22 (Sinonasal Outcome Test-22) scale was applied by the researcher to the patients on the day of admission [10]. Lund-Mackay (LM) scoring was calculated by examining the Paranasal CT images of the patients [11]. The control group consisted of healthy volunteers.

In total 18 CRSwNP patients and 10 healthy individuals participated in the study. Participants’ demographic information, SNOT-22 and LM scores were summarized in Table 1. The groups have similar demographic characteristics.

**Table 1:** Demographic information, SNOT-22, and LM scores of the participants.

		Primary Disease	Recurrent Disease	Control Group	Overall	p
**n**		10	8	10	28	
**Age**		36.6±11.7	40.6±11.9	34.4±9.7	36.96±11.0	0.49
**Sex**	**M**	6(60%)	5(62.5%)	7(70%)	18(64.3%)	0.89
	**F**	4(40%)	3(37.5%)	3(30%)	10(35.7%)	
**Asthma**		3 (%30)	2 (%25)	0	5 (17.9%)	0.18
**Smoking**		3 (30%)	3 (37.5%)	4 (40%)	10 (35.7%)	0.89
**SNOT-22**		42.6±19.5	32.0±11.4	N/A	37.9±16.9	0.10
**LMS**		18.6±4.7	16.5±5.1	N/A	17.7±4.8	0.41
**LMS-Sampling side**		9.3±2.3	8.6±2.7	N/A	9.0±2.5	0.69

### Sample gathering

A swab sample was taken from the middle meatus from each patient under endoscopic examination to prevent contamination. Samples were taken using a sterile cotton tipped swab. The sample was taken by rotating the swab tip (at least 10 turns) until it is visibly saturated. Swab tips were cut and transferred to a sterile container. The samples were transferred into the -80ºC freezer as soon as possible and kept at -80ºC until the DNA extraction procedure [12].

### DNA extraction

DNA extraction was performed with QIAamp DNA mini kit (Qiagen, Germany). The samples were taken out of the -80ºC freezer and thawed at room temperature for one hour. The swab tips were divided into small pieces with help of sterile scalpels and forceps. 180 μL of enzymatic solution and 20 μl of Proteinase K were added and mixture incubated at 56ºC overnight. Consequent steps were followed according to the instructions in the manufacturer’s manual. In addition, the elution step was done with 50 μL solution instead of 250 μL. DNA was preserved at -20 ºC until further processing [13].

### Next Generation Sequencing

Libraries were constructed from double-stranded cDNA using Swift Biosciences’. Swift Amplicon 16S+ITS Panel (Swift Biosciences) was used as single pool target enrichment for metagenomics by Next Generation Sequencing (NGS). Swift Amplicon 16S+ITS panel profiles complex metagenomics samples and leverages multiplexed primers covering all variable regions of 16S rRNA, ITS1 and ITS2, in one PCR reaction. They were sequenced on MiSeq platform (Illumina) using the 2 × 250 bp paired-end protocol. These sequence data have been submitted to the GenBank databases under accession number SUB9604990.

### Bioinformatic and statistical analysis

Illumina paired end reads were joined using fastq-join and then filtered with QIIMEI’s split_libraries_fastq.py workflow script using the QIIME software package [14]. Briefly, both 16S rRNA gene and ITS region sequences were separately filtered, assembled, trimmed, and controlled for quality. Downstream analyses of quality and chimera filtered reads were performed. Quality filtered sequencing read datasets were >250 bp and quality scores were > 30 PHRED. Samples were separately assigned to operational taxonomic units (OTUs) with a threshold of 97% pairwise identity using QIIMEI’s reference-based workflow scripts and SILVA v138 database [15] for 16S-rRNA and Unite v8.2 database [16] for ITS.

IBM SPSS Statistic v22 was used for statistical analysis. *p<*0.05 was considered significant for all comparisons. Mann Whitney U or Kruskal Wallis tests were used to compare mean relative abundance of taxa between groups. Spearman Correlation Coefficient was used to evaluate the degree of correlation between two variables. The alpha diversity of the microbial community profile in the samples was tested for significance through Kruskal-Wallis (pairwise) analysis. Jaccard distance, and Bray Curtis dissimilarity analysis were applied to assess the beta diversity. The beta diversity of the microbial community profile between samples was tested for significance through Permutational Multivariate Analysis of Variance (PERMANOVA). Linear Discriminant Analysis (LDA) and Linear Discriminant Analysis Effect Size (LEfSe) was further analyzed to define the potential biomarkers with differences in abundance between the samples [17].

## Results

### Diversity

The median values of the Shannon diversity index in the primary disease, recurrent disease and control groups were 6.8, 6.3 and 5.4, respectively (p = 0.190) and the median values of Faith’s phylogenetic diversity (FPD) index in primary disease, recurrent disease and control groups were 46.8, 47.8 and 37.8, respectively (p = 0.377).

**Fig 1:**
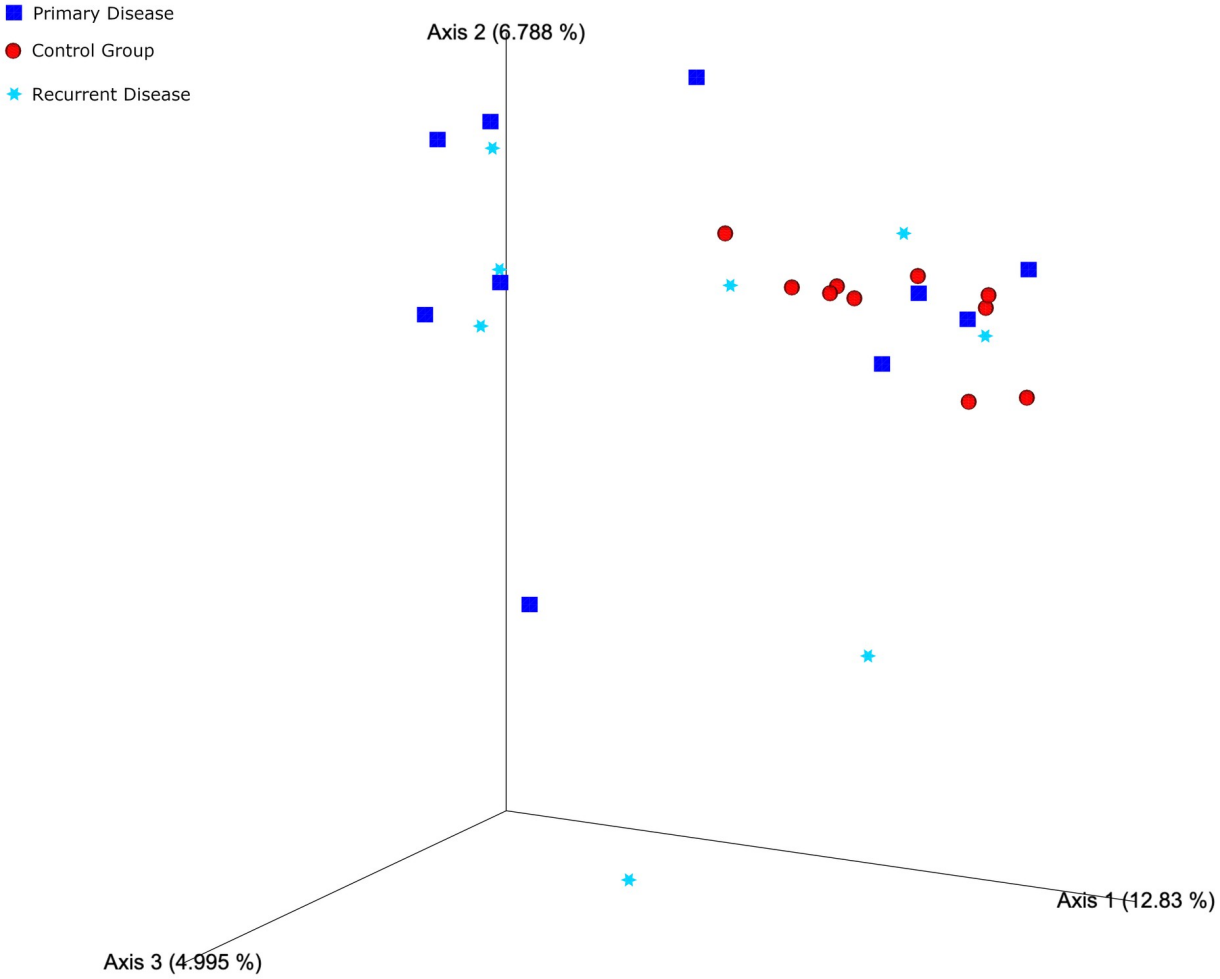
Beta diversity of nasal microbiota illustrated in PCoA based on Jaccard distance index (PCoA: Principal Coordinate Analysis, CRSwNP: Chronic Rhinosinusitis with nasal polyposis).

Analysis of Bray-Curtis dissimilarity showed a statistically significant difference between the beta diversity of the control group and the primary disease group (p = 0.009) There was no significant difference between the control and recurrent disease groups and between recurrent disease and primary disease groups (p = 0.055 and p = 0.261, respectively). There was a significant difference in beta diversity between CRSwNP patients and control group (p = 0.012). Based on Jaccard distance, the beta diversity of the control group was found to be significantly different from the primary disease and recurrent disease groups (p = 0.008 and p = 0.005, respectively). There was no difference between primary disease and recurrent disease groups (p = 0.726). In Principal Coordinate analysis (PCoA) plots based on Bray-Curtis dissimilarity and Jaccard distance, subjects in CRSwNP groups showed higher degrees of inter-subject variability compared to control group (Fig 1).

### Bacterial composition

A total of 25 different phyla were detected in the samples. According to mean relative abundance (MRA) the predominant phyla were Firmicutes (40.4%), Actinobacteria (38.7%) Proteobacteria (16.2%) for the control group, Firmicutes (41.0%) Proteobacteria (31.5%), Actinobacteria (19.4%) for the primary disease group, and Firmicutes (53.9%), Proteobacteria (28.6%), Actinobacteria (13.4%) for the recurrent disease group.

A total of 518 genera were detected in the samples. The predominant genera according to MRA were *Staphylococcus* (22.2%), *Corynebacterium* (16.2%), *Sphingomonas* (9.2%) for all participants, *Corynebacterium* (30.5%), *Staphylococcus* (24.0%), *Sphingomonas* (7.7%) for control group, *Streptococcus* (11.5%), *Sphingomonas* (11.2%), *Corynebacterium* (10.5%) for primary disease group, *Staphylococcus* (37.8%) *Sphingomonas* (8.6%), *Corynebacterium* (5.4%) for the recurrent disease group, and *Staphylococcus* (21.3%), *Sphingomonas* (10.0%), *Streptococcus* (8.3%) for all CRSwNP patients. Prevalence and MRA of 32 the most abundant genera were summarized in Table 2.

**Table 2:** Mean relative abundance and prevalence of genus-level bacteria by groups.

Genera	Control Group		Primary Disease		Recurrent Disease		All CRSwNP		Overall	
	MRA (%)	Prevalence (%)	MRA (%)	Prevalence (%)	MRA (%)	Prevalence (%)	MRA (%)	Prevalence (%)	MRA (%)	Prevalence (%)
*Staphylococcus*	24.0	100.0	8.1	100.0	37.8	100.0	21.3	100.0	22.2	100.0
*Corynebacterium*	30.5	100.0	10.5	100.0	5.4	100.0	8.2	100.0	16.2	100.0
*Sphingomonas*	7.7	100.0	11.2	90.0	8.6	87.5	10.0	88.9	9.2	92.9
*Streptococcus*	2.0	100.0	11.5	100.0	4.3	100.0	8.3	100.0	6.1	96.4
*Bacillus*	3.6	100.0	4.1	50.0	4.9	50.0	4.5	50.0	4.2	67.9
*Pseudomonas*	3.0	100.0	5.4	90.0	2.2	87.5	4.0	88.9	3.6	92.9
*Cutibacterium*	4.5	100.0	2.9	100.0	2.6	100.0	2.7	100.0	3.4	100.0
*Parvimonas*	0.1	30.0	7.8	60.0	0.0	50.0	4.3	55.6	2.8	46.4
*Lawsonella*	2.8	100.0	2.0	100.0	0.9	87.5	1.5	94.4	2.0	96.4
*Dolosigranulum*	4.0	50.0	0.6	50.0	0.0	50.0	0.3	50.0	1.7	50.0
*Peptoniphilus*	2.2	90.0	1.1	90.0	1.6	87.5	1.3	88.9	1.6	89.3
*Anaerococcus*	2.0	100.0	1.6	90.0	0.9	87.5	1.3	88.9	1.5	92.9
*Moraxella*	0.0	30.0	0.2	70.0	5.0	75.0	2.3	72.2	1.5	57.1
*Rothia*	0.3	80.0	1.9	90.0	2.3	100.0	2.1	94.4	1.5	89.3
*Acinetobacter*	0.4	100.0	2.1	90.0	1.4	90.0	1.8	88.9	1.3	92.9
*Pasteurellaceae_unclassified*	0.1	60.0	1.7	100.0	2.1	100.0	1.9	100.0	1.3	85.7
*Neisseria*	0.1	90.0	1.3	90.0	2.1	87.5	1.7	88.9	1.1	89.3
*Haemophilus*	0.1	90.0	1.2	100.0	2.0	62.5	1.6	83.3	1.1	85.7
*Prevotella*	0.8	90.0	1.0	90.0	0.8	100.0	0.9	94.4	0.9	92.9
*Bdellovibrio*	0.8	100.0	1.1	80.0	0.5	50.0	0.8	66.7	0.8	78.6
*Fusobacterium*	1.1	60.0	0.7	90.0	0.5	75.0	0.6	83.3	0.8	75.0
*Asticcacaulis*	0.6	100.0	0.9	70.0	0.5	62.5	0.7	66.7	0.7	78.6
*Peptostreptococcus*	0.1	30.0	1.7	60.0	0.1	100.0	1.0	77.8	0.7	60.7
*Enhydrobacter*	0.2	90.0	0.8	80.0	0.8	100.0	0.8	88.9	0.6	89.3
*Granulicatella*	0.1	50.0	1.0	90.0	0.6	50.0	0.8	72.2	0.6	64.3
*Lactobacillus*	0.2	90.0	0.8	90.0	0.6	100.0	0.7	94.4	0.5	92.9
*Finegoldia*	0.6	90.0	0.5	100.0	0.5	75.0	0.5	88.9	0.5	89.3
*Actinomyces*	0.1	100.0	0.8	100.0	0.5	75.0	0.7	88.9	0.5	92.9
*Micrococcus*	0.1	70.0	0.5	90.0	0.5	75.0	0.5	83.3	0.4	78.6
*Brevundimonas*	0.4	100.0	0.4	60.0	0.3	87.5	0.3	62.2	0.4	82.1
*Porphyromonas*	0.7	60.0	0.2	90.0	0.1	75.0	0.2	83.3	0.4	75.0

Mean relative abundance of *Corynebacterium* was found to be significantly higher in the control group compared to the patients (p = 0.007). MRAs of *Streptococcus*, *Moraxella*, *Prevotella*, *Pasteurellaceae unclassified*, *Peptostreptococcus and Micrococcus* were significantly higher in the patient groups compared to the control group (p = 0.025, p = 0.015, p = 0.040, p = 0.001, p = 0.045, p = 0.021, respectively).

**Fig 2:**
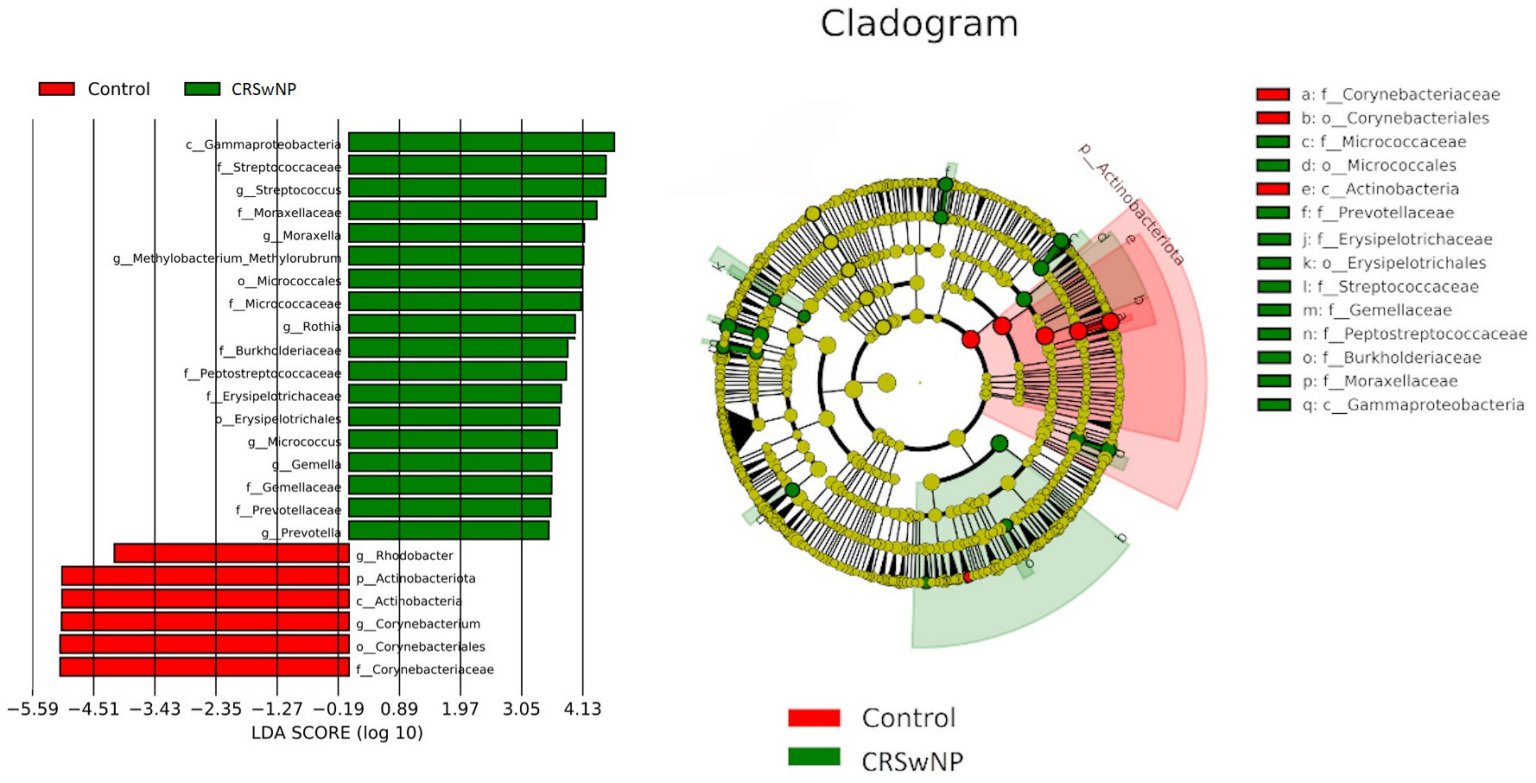
Comparison of nasal microbiota composition with LEfSe: A: Histogram of the LDA scores computed for features differentially abundant between Control group and CRSwNP patients B: Taxonomic representation of statistically and biologically consistent differences between Control group and CRSwNP patients. Differences are represented in the color of the most abundant class (red indicating control, green CRSwNP, yellow non-significant). Each circle’s diameter is proportional to the taxon’s abundance. (LDA: linear discriminant analysis, LEfSe: linear discriminant analysis effect size, CRSwNP: Chronic Rhinosinusitis with nasal polyposis).

LEfSe comparison of bacterial communities of CRSwNP patients and control group highlighted *Corynebacterium*, as the differentiating taxa for control group and *Streptococcus*, *Moraxella*, *Rothia*, *Micrococcus*, *Gemella*, and *Prevotella* for CRSwNP patients (Fig 2).

### Fungal composition

**Fig 3:**
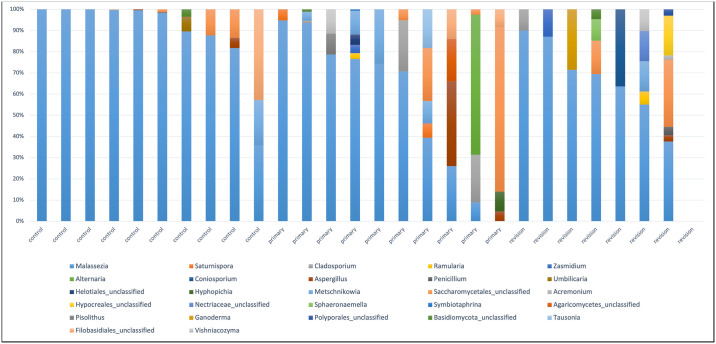
Relative abundance of genus-level fungi for each sample.

Fungal phyla identified in the samples were Basidiomycota and Ascomycota. MRA of Basidiomycota was 75.8% in the control group, 41.2% in the primary disease group, and 26.8% in the recurrent disease group. A total of 29 fungal genera were detected in the samples. The most abundant fungus in all groups was Malassezia (Fig 3). MRA of Malassezia was found as 89.2% in the control group, 56.3% in the primary disease group, and 59.3% in the recurrent disease group. MRA in the control group was significantly higher than the patient groups (p = 0.001).

### Disease severity

**Fig 4:**
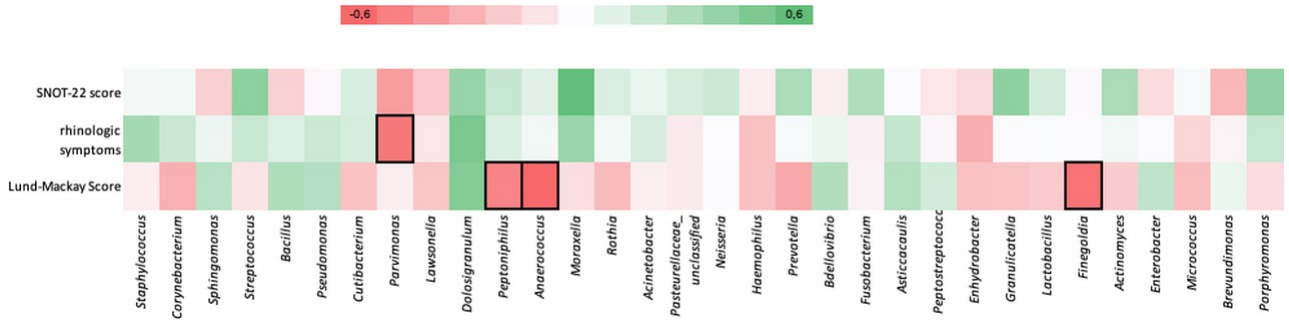
Correlation of relative abundances of genus-level bacteria with SNOT-22 and Lund Mackay scores. Marked boxes represent a statistically significant Spearman correlation.

A negative correlation was observed between the relative abundance of *Peptoniphilus*, *Anaerococcus* and *Finegoldia* and Lund Mackay scores (p = 0.005, p = 0.001, p = 0.002, respectively). Parvimonas showed a negative correlation with the total score obtained from the first 12 items of the SNOT-22 scale in which rhinologic symptoms are questioned (p = 0.01). The correlation between the relative abundance of bacteria at the genus level and the SNOT-22 and Lund Mackay scores is shown in Fig 4 with the heat map conducted according to the Spearman correlation coefficient.

### Correlation between taxa

**Fig 5:**
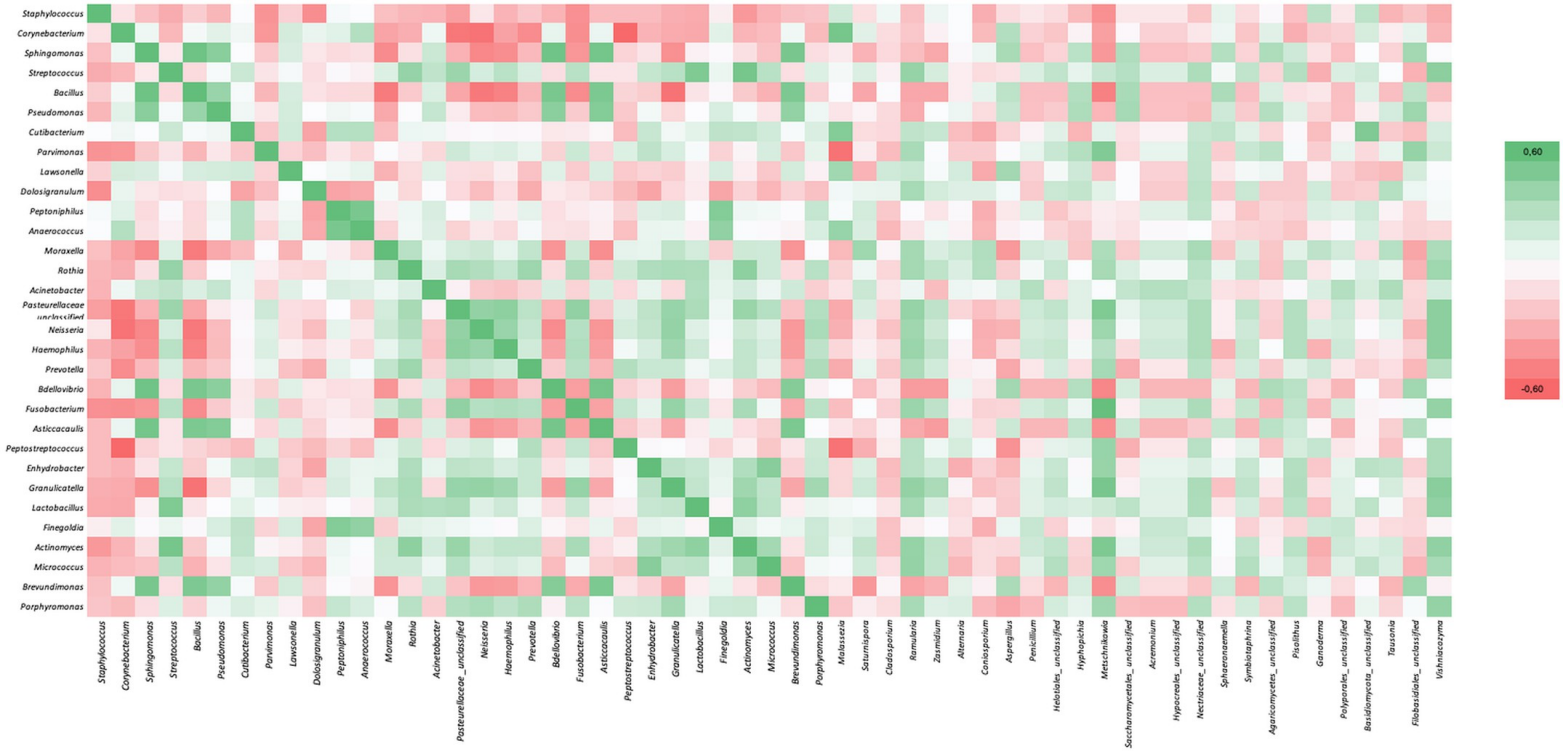
Correlation of OTU counts of genus-level bacteria with each-other and with genus-level fungi based on the Spearman correlation analysis (OTU: Operational taxonomic unit).

The correlation of OTU counts of bacteria with other bacteria and fungi was investigated at genus level (Fig 5). *Staphylococcus* showed a statistically significant negative correlation with *Dolosigranulum* and *Fusobacterium* (p <0.05). *Corynebacterium* had a positive correlation with *Lawsonella* and *Anaerococcus*, and a negative correlation with *Neisseria*, *Prevotella*, *Fusobacterium*, *Pasteurellaceae*_*unclassified* and *Peptostreptococcus* (p <0.05). *Malassezia* correlated positively with *Corynebacterium*, *Cutibacterium* and *Anaerococcus* and negatively with *Parvimonas* and *Peptostreptococcus* (p <0.05). Aspergillus had negative correlation with *Moraxella* and *Peptostreptococcus* (p <0.05).

## Discussion

The role of microbial factors in the pathogenesis of CRSwNP has not yet been clarified and is still an important research topic. Advances in the field of NGS enable microbiome research. In this study, we investigated how the bacterial and fungal composition in the nasal microbiome differed between the healthy control group and CRSwNP patients.

Alpha diversity refers to the diversity of species within a local habitat (single individual for microbiome studies) [18] Choi et al. [9] and Chalermwatanachai et al. [19] reported decreased alpha diversity in CRSwNP patients compared to the control group and claimed that decreased alfa diversity could have a role in pathogenesis. On the other hand, Copeland et al. [20] and Cleland et al. [21] stated that there was no difference in alpha diversity between the control and CRSwNP patients. In current study, alfa diversity did not differ significantly between groups.

Beta diversity refers to the diversity between different habitats (each individual in microbiome studies) [22]. A significant difference was observed between beta diversity of control group and CRSwNP patients. In the PCoA plots based on both indices, it was seen that the individuals in the control group clustered closer to each other, and CRSwNP patients were located more distant from each other and the control group. In the same line with our study, Ramakrishnan et al. [23], and Hoggard et al. [24], reported higher inter-individual variations in CRSwNP patients compared the control group according to the PCoA plots conducted with beta diversity indices. These findings indicate that CRSwNP is caused by a change in microbial composition.

LEfSe is an algorithm for high-dimensional biomarker discovery and description that defines genomic features (genes, pathways, or taxa) that characterize differences between two or more biological classes. With emphasizing statistical significance, biological consistency and effect relevance, LEfSe allows researchers to identify differentially abundant taxa that are also consistent with biologically meaningful categories [17]. LefSE analysis showed that *Streptococcus*, *Moraxella*, *Rothia*, *Micrococcus*, *Gemella and Prevothelia* are differentiating taxa In the CRSwNP group. In addition, *Streptococcus*, *Moraxella*, *Prevotella*, *Peptostreptococcus*, *Micrococcus*, *and Pasteurellaceae_unclassified* had higher MRA in CRSwNP patients compared to the control group. Gan et al. [25] stated *Lactobacillus*, *Streptococcus*, *Moraxella*, *Haemophilus*, *Fusobacterium and Prevotella* showed higher MRA in CRSwNP patients compared to the control group. *Streptococcus*, *Moraxella*, *Prevotella*, *Peptostreptococcus* are frequently isolated microorganisms in culture studies with sinus aspirates of CRS patients [26]. These findings are consistent with the polymicrobial nature of CRS etiopathogenesis.

Ramakrishnan et al. [23] reported that the higher MRA of *Corynebacterium* preoperatively increased the surgical success in CRS patients and the excess of Staphylococcus was associated with a poorer surgical outcome. Among the groups in our study, the lowest MRA of *Corynebacterium* and the highest MRA of *Staphylococcus* were observed in the recurrent disease group. Although the microbial composition of the recurrent disease group before surgery is not known due to the cross-sectional structure of current study, it may be thought that increased *Staphylococcus* and decreased *Corynebacterium* abundance may be associated with recurrence. On the other hand, previous endoscopic sinus surgery may have caused this change in microbiome composition. In a previous longitudinal study, Jain et al. [27] reported the genera with the highest MRA in CRS patients were *Streptococcus*, *Corynebacterium*, *Haemophilus* and *Staphylococcus*, respectively in preoperative period and *Staphylococcus*, *Corynebacterium*, *Haemophilus* and *Streptococcus*, respectively in postoperative period. This finding may explain the difference in microbial composition between the primary disease group and the recurrent disease group. In our study, in accordance with this finding, genus with the highest MRA in the primary disease group was *Streptococcus*, while genus with the highest MRA in the recurrent disease group was *Staphylococcus*.

Before the use of 18S rRNA and ITS sequencing techniques, fungal isolation and identification in the nasal cavity and paranasal sinuses were performed by methods such as culture and microscopy, and fungal isolation was not successful in many cases [28]. *Malassezia* was ignored in isolations made with classical methods due to the difficulties in cultivation [29]. Gelber et al. [30] were able to isolate Malassezia in healthy individuals and CRS patients with qPCR method and emphasized that Malassezia was commensal; however, Malassezia was isolated in only 68% of the participants. In two studies using new generation sequencing methods (one study sequenced ITS2 and the other sequenced 18S rRNA), Malassezia was stated to be the predominant fungus in the nasal cavity and paranasal sinuses and was isolated in all of the participants in both studies [31, 32]. Another study investigating fungi in the nasal microbiome with ITS1 sequencing indicated Aspergillus as the most predominant fungus genus, but in this study, no fungus could be isolated from 44.4% of the participants [33].

Nasal microbiome could be related to disease severity of CRSwNP patients. Copeland et al. [20] stated that SNOT-22 scores were positively correlated with the MRA of Escherichia and negatively correlated with the MRA of Corynebacterium. Mahdavinia et al. [34] reported a significant negative correlation between the MRA of Prevotella and SNOT-22 scores. Cleland et al. [21] stated that the MRA of Pseudomonas was associated with higher SNOT-22 scores. In our study, no bacterial species that showed a significant correlation with the SNOT-22 score was detected. The MRA of Parvimonas had negative correlation with the score of the first 12 questions of the SNOT-22 scale which cover rhinosinusitis symptoms. Abbas et al. [35] reported that the LM scores of CRSwNP patients showed positive correlation with MRA of *Moraxella*, *Parvimonas* and negative correlation with the MRA of *Succinivibrio* and *Exiguobacterium*. Mahdavinia et al. [34] found a positive correlation between the MRA of *Enterobacteriaceae* and LM score. In our study, a negative correlation was found between the MRAs of *Peptoniphilus*, *Anaerococcus*, *Finegoldia* and LM scores.

It is known that microorganisms within the microbiome can affect each other competitively or cooperatively. Correlation matrix is a simple method to evaluate the interaction of microorganisms. *Corynebacterium* showed negative correlation with microorganisms frequently isolated in the purulent discharge of CRS patients such as *Prevotella*, *Fusobacterium* and *Peptostreptococcus*. *Corynebacterium* was the differentiating taxa in the control group according to biomarker research with LEfSe analysis. Thus, some species in genus *Corynebacterium* may have a regulatory function in the nasal microbiome. Some *Corynebacterium* genus bacteria are in a competitive relationship with S. aureus, for example, *Corynebacterium pseudodiphtheriticum* has been shown to act as a probiotic in the elimination of nasal *Staphylococcus aureus* colonization [36, 37]. Copeland et al. [20] investigated the interactions of bacteria with each other and their relationship with disease severity; They stated that *Dolosigranulum* and *Corynebacterium* were positively correlated with each other and both were negatively correlated with disease severity. Gan et al. [25] stated that the MRA of *Corynebacterium and Dolosigranulum* in the control group was higher than in CRSwNP patients. Brugger et al. [38] demonstrated in vitro that *Dolosigranulum pigrum* inhibits the growth of *S*. *aureus*. In our study, *Dolosigranulum* had a higher MRA in the control group compared to the patient groups and was also positively correlated with *Corynebacterium*, and negatively correlated with *Staphylococcus*. On the other hand, *Dolosigranulum* was associated with higher LM and SNOT-22 scores in current study. In addition, its prevalence was low in both the control and patient groups. We could not find enough data to evaluate its regulatory role in the microbiome and its protective effect.

*Malassezia* was positively correlated with *Corynebacterium* and *Anaerococcus*. Similarly, Hoggard et al. [31] also stated that *Malassezia* was positively correlated with *Corynebacterium* and *Anaerococcus*. It may be thought that some species of genus *Malassezia* may be protective against CRSwNP due to its positive correlation with *Corynebacterium* and significantly higher MRA in the control group.

This study used a novel method with performing 16S rRNA and ITS next-generation sequencing on the same sample with a single kit in CRSwNP patients. This made a significant contribution to our study in terms of evaluating the bacterial-fungal interaction. In addition, sequencing of all variable regions between the V1-V9 regions of 16s rRNA increased the power of the study. The interactions between species may lead to new treatment options like probiotics.

There were a few limitations in our study that should be emphasized. Firstly, due to the high cost of the study, a small number of participants could be included in the study. Secondly, we investigated the microbiome comparison between patients with recurrence after previous sinus surgery and patients who did not receive surgical treatment by designing a cross-sectional study. A longitudinal study that investigates the microbiome before endoscopic sinus surgery and in the long term would be more effective to evaluate the effect of surgery. In addition, microbiome studies conducted with 16s rRNA next-generation sequencing have some limitations. It has been stated that the results may vary significantly depending on the DNA extraction method used [39] In addition, DNA is obtained from non-living microorganisms during DNA extraction. Furthermore, bacteria’s 16s rRNA gene copies are variable, resulting in an increased abundance of high copy number species [40]. The sequencing analysis part may also cause significant changes in the results. To avoid overestimating microbial diversity, analysis should be performed with the highest quality sequences possible [41]. Additionally, the databases used in taxonomic classification may also affect the results [40]. Extraction and analysis of metagenomic mRNA may be a more accurate method to assess the functional and metabolic diversity of microbial communities [19].

## Conclusion

Dysbiosis of the nasal microbiome has a role in the pathogenesis of CRSwNP. Nasal microbiome of CRSwNP patients shows greater inter-individual variation than the control group. Some species of the genus *Corynebacterium* may have regulatory function in microbiome to prevent development of the disease. *Malassezia* is the dominant fungus genus in the nasal cavity and paranasal sinuses.

## Supporting information

S1 File (XLSX)

S1 Data (SAV)

S2 Data (SAV)
